# Fibrinogen-to-Albumin Ratio and Long-Term Mortality in Coronary Artery Disease Patients with Different Glucose Metabolism Status

**DOI:** 10.31083/j.rcm2411317

**Published:** 2023-11-16

**Authors:** Yun Xie, Xiayan Xu, Dongmei Wang, Yang Zhou, Yu Kang, Wenguang Lai, Hongyu Lu, Jin Liu, Shiqun Chen, Junyan Xu, Xiaoming Yan, Xiaoyu Huang, Yong Liu

**Affiliations:** ^1^School of Biology and Biological Engineering, South China University of Technology, 510006 Guangzhou, Guangdong, China; ^2^Department of Cardiology, Guangdong Cardiovascular Institute, Guangdong Provincial People’s Hospital (Guangdong Academy of Medical Sciences), Southern Medical University, 510080 Guangzhou, Guangdong, China; ^3^Department of Guangdong Provincial Key Laboratory of Coronary Heart Disease Prevention, Guangdong Cardiovascular Institute, Guangdong Provincial People’s Hospital (Guangdong Academy of Medical Sciences), Southern Medical University, 510080 Guangzhou, Guangdong, China; ^4^School of Medicine, South China University of Technology, 510006 Guangzhou, Guangdong, China; ^5^Guangdong Provincial People’s Hospital, Guangdong Academy of Medical Sciences, Guangdong Cadres Health Management Center, 510080 Guangzhou, Guangdong, China; ^6^Shantou University Medical College, 515041 Shantou, Guangdong, China; ^7^Department of Information Technology, Guangdong Provincial People's Hospital (Guangdong Academy of Medical Sciences), Southern Medical University, 510080 Guangzhou, Guangdong, China; ^8^Department of Cardiology, Yangjiang People’s Hospital, 529500 Yangjiang, Guangdong, China

**Keywords:** fibrinogen-to-albumin ratio, diabetes, prediabetes, long-term mortality, coronary artery disease

## Abstract

**Background:**

Abnormal glucose metabolism is present in most patients with coronary artery disease (CAD). Inflammation is considered to be a common risk factor for CAD and diabetes. Fibrinogen-to-albumin ratio (FAR), a novel inflammation biomarker, has been proposed as a predictor for cardiovascular disease. However, the relationship between the level of FAR and long-term mortality including all-cause, cardiovascular and cancer mortality, remains unknown in CAD patients, especially those with prediabetes.

**Methods:**

We enrolled 66,761 CAD patients from 2007 to 2020 from a multi-center registry cohort study. The primary outcomes were the all-cause, cardiovascular and cancer mortality. FAR was calculated using the following formula: Fibrinogen (g/L)/Albumin (g/L). Patients were divided into three groups by FAR tertile (low FAR (FAR-L), median FAR (FAR-M), high FAR (FAR-H)), and further categorized into 9 groups according to FAR and glucose metabolism status (normal glucose regulation (NGR), prediabetes mellitus (PreDM), diabetes mellitus (DM)). Cox regression models and competing risk models were used to examine the relationships between FAR and clinical outcomes.

**Results:**

66,761 patients (63.1 ± 11.0 years, 75.3% male) were enrolled. During the follow-up, 10,534 patients died, including 4991 cardiovascular deaths and 1092 cancer deaths. After adjusting for confounders, higher FAR was associated with increased risk of all-cause and cause-specific mortality in CAD patients with NGR, PreDM and DM. The risk of all-cause and cardiovascular mortality was highest in FAR-H with DM (HR (95% CI) = 1.71 (1.58–1.86), 2.11 (1.86–2.38), respectively; *p* < 0.001). FAR-H with PreDM was significantly associated with the highest risk of cancer mortality (HR (95% CI) = 2.27 (1.70–3.02), *p* < 0.001). Adding FAR to the original model significantly improved the prediction of long-term mortality.

**Conclusions:**

Increased FAR was significantly associated with higher risk of all-cause and cause-specific mortality in CAD patients with NGR, PreDM and DM. Abnormal glucose metabolism augments the relationship between FAR and mortality.

**Clinical Trial Registration:**

ClinicalTrials.gov NCT05050877.

## 1. Introduction

Coronary artery disease (CAD) is identified as a major cause of death with more than 9 million deaths in 2019, accounting for 16% of all-cause deaths worldwide [1]. Despite the significant advancements in medical treatment, patients with CAD still face a substantial risk of death, which imposes immense health and economic burdens worldwide [2].

Abnormal glucose metabolism, including diabetes mellitus (DM) and prediabetes mellitus (PreDM), is present in most patients with CAD [3]. The 2019 ESC Guidelines on diabetes, prediabetes, and cardiovascular diseases indicate that about 20–30% of patients with CAD have DM, and up to 70% of the remaining patients will be newly diagnosed with DM or PreDM [4]. Multiple epidemiological studies have confirmed that DM is one of the risk factors for CAD patients [5, 6]. In addition, PreDM also increases the risk of adverse prognosis in CAD patients [7, 8]. As a result, it is critical to pay closer attention to the glucose metabolism status of CAD patients in order to stratify their risk and better manage these risk factors for patients who are at high risk.

Inflammation is considered to be a common risk factor for CAD and diabetes [9, 10]. A number of studies have shown that inflammatory markers have increased sensitivity for predicting the prognosis of both diabetes and CAD [11, 12, 13, 14]. Fibrinogen (FIB), an inflammatory marker synthesized by the liver, as well as a coagulation factor involved in the formation of thrombosis and the progression of atherosclerosis, has been found to be closely related to the poor prognosis of CAD patients [15, 16]. Albumin (ALB) is the most abundant plasma protein and has anti-inflammatory, antioxidant, and antithrombotic properties. Previous studies have indicated that low serum albumin concentration is associated with an increased risk of adverse cardiovascular events in CAD patients [17, 18]. Fibrinogen-to-albumin ratio (FAR) is an innovative inflammatory biomarker that combines the above two indicators and has been widely used to predict adverse prognosis among patients with various cancers [19, 20]. Recently, several studies have also confirmed that FAR is strongly linked to the severity of coronary lesions and poor clinical outcomes in patients with ST-segment elevation myocardial infarction (STEMI) and multivessel disease [21, 22, 23]. However, the relationship between FAR, all-cause and cause-specific mortality is not fully understood in CAD patients with different glucose metabolism, especially those with prediabetes.

The purpose of this study was to investigate the potential relationship between FAR and long-term mortality among CAD patients, including all-cause, cardiovascular and cancer mortality, and further determine whether the association between FAR levels and clinical outcomes varies according to the glucose metabolism status, especially in those patients with prediabetes.

## 2. Materials and Methods

### 2.1 Study Population

**Fig. 1.**
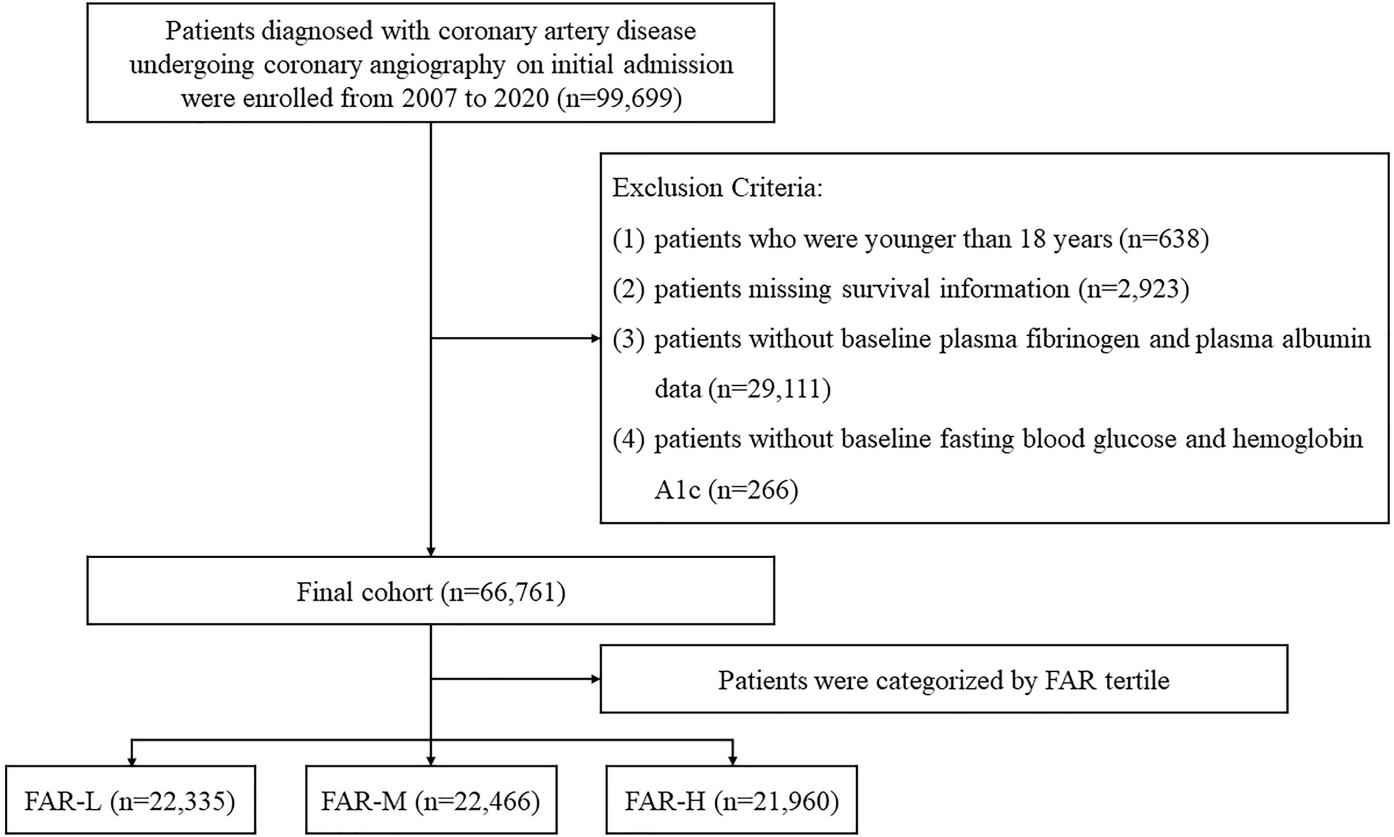
**Flow of patients through the study**. FAR, fibrinogen-to-albumin ratio; FAR-L, low FAR; FAR-M, median FAR; FAR-H, high FAR.

This multi-center cohort study was based on the Cardiorenal Improvement II (CIN-II) study, which included patients recruited from 5 large tertiary hospitals in China between January 2007 and December 2020 (Cardiorenal Improvement II, ClinicalTrials.gov NCT05050877). We enrolled 66,761 CAD patients who underwent coronary angiography (CAG) at the time of initial admission. The exclusion criteria were as follows: (1) patients younger than eighteen years; (2) patients missing survival information; (3) patients without baseline plasma fibrinogen and plasma albumin data; (4) patients without baseline fasting blood glucose (FBG) and hemoglobin A1c (HbA1c) (Fig. 1). This research was approved by the Ethics Committee of the Guangdong Provincial People’s Hospital (No. GDREC2019-555H-2). To protect the privacy of patients, we removed all traceable personal identifiers from the database, and each participating site obtained institutional review board permission from the local ethics committees. Since this research involved retrospective cases, no additional intervention was necessary. Furthermore, the data we used has been desensitized and patient informed consent was not required. The study complied with the Declaration of Helsinki.

### 2.2 Baseline Data Collection

The baseline information including demographic characteristics, complications, procedures, laboratory examinations, and medications were obtained from the electronic clinical management system (ECMS). On initial admission, biochemistry data such as plasma fibrinogen, plasma albumin and HbA1c were obtained. Survival information was derived from a cause-specific surveillance dataset at the regional Center for Disease Control and Prevention (CDC).

### 2.3 Study Outcomes and Clinical Definition

The outcomes of this study were all-cause, cardiovascular and cancer mortality. FAR was defined as the ratio of fibrinogen (g/L) to albumin (g/L). According to the American Diabetes Association [24], DM was identified by FBG ≥7.0 mmol/L (126 mg/dL), or HbA1c ≥6.5%, or 2-h blood glucose of oral glucose tolerance test ≥11.1 mmol/L (200 mg/dL), or a previous diagnosis of DM with antidiabetic treatment. PreDM was diagnosed by 5.6 mmol/L ≤ FBG < 7.0 mmol/L or 5.7% ≤ HbA1c < 6.5%. Normal glucose regulation (NGR) was defined as patients without PreDM or DM. The Chronic Kidney Disease Epidemiology Collaboration (CKD-EPI) equation was used to calculate the estimated glomerular filtration rate (eGFR), and chronic kidney disease (CKD) was defined as eGFR 60 mL/min/1.73 $m^{2}$ [25, 26]. Hyperlipemia, acute myocardial infarction (AMI) and hypertension (HT) were defined by using the 10th Revision Codes of the International Classification of Diseases (ICD-10) codes. Congestive heart failure (CHF) was defined as New York Heart Association class ≥3 or Killip class >1.

### 2.4 Statistical Analysis

According to the baseline FAR, patients were categorized into three groups: FAR-L, FAR-M, FAR-H, and further divided into nine groups according to FAR and glucose metabolism status (NGR, PreDM, DM). For the baseline characteristics, continuous variables were presented as mean with standard deviation (SD) or median with interquartile range (IQR) as appropriate. Categorical variables were described as counts and percentages. One-way ANOVA or the Kruskal–Wallis test was used for continuous variables. The χ^2^ test was used for categorical variables. In order to examine the correlation between FAR levels as a continuous variable and mortality for all-cause, cardiovascular, and cancer, we conducted restricted cubic splines (RCS) analyses. Kaplan-Meier survival curves were used to describe the time-to-event data, and differences were assessed using the log-rank tests. We evaluated the relationship between FAR and all-cause mortality by Cox regression models. FAR and cause-specific mortality were assessed by competing-risk models. Models were adjusted for the following covariates: age, gender, HT, CHF, AMI, CKD, stroke, hyperlipemia, respectively. A variance inflation factor ≥5 indicates the presence of multicollinearity between variables. The C-index was constructed to evaluate the change in the predictive accuracy of long-term mortality after the addition of FAR to the original clinical risk factors model (age, gender, HT, CHF, AMI, CKD, stroke, hyperlipemia). Correlations between HbA1c, FBG, FAR and its components were assessed using the Spearman correlation test. All data were analyzed using R version 4.0.3. (R Foundation for Statistical Computing, Vienna, Austria). A two-sided *p* value < 0.05 was considered statistically significant.

## 3. Results

### 3.1 Baseline Characteristics

A total of 66,761 patients with CAD (the mean age 63.1 ± 11.0 years, 75.3% were men) were enrolled in this study, including 21,982 patients with NGR, 20,723 with PreDM and 24,056 patients with DM. Based on the baseline FAR, the patients were categorized into 3 groups: FAR-L group (n = 22,335), FAR-M group (n = 22,466), FAR-H group (n = 21,960). The baseline characteristics between patients with different FAR are presented in Table 1. The FAR-M group had the highest proportion of females. From the FAR-L to the FAR-H group, patients with higher FAR were more likely to have HT, CHF, CKD, hyperlipemia and anemia. In addition, they had higher levels of eGFR, HbA1c, FIB, and lower levels of HGB, HDLC and ALB.

**Table 1.** **Baseline characteristics based on FAR levels**.

			Overall	FAR-L	FAR-M	FAR-H	*p* value
			n = 66,761	n = 22,335	n =22,466	n = 21,960	
Demographic characteristics							
	Age, years		63.1 (11.0)	61.4 (11.2)	63.7 (10.5)	64.5 (10.9)	<0.0001
	Age >60, n (%)		40,666 (60.8)	12,361 (54.2)	14,437 (63.3)	13,823 (65.4)	<0.0001
	Female, n (%)		16,514 (24.7)	5086 (22.3)	6239 (27.4)	5170 (24.4)	<0.0001
Complication							
	Glucose metabolism status						<0.0001
		NGR, n (%)	21,982 (32.9)	9439 (42.3)	6825 (30.4)	5715 (26.0)	
		PreDM, n (%)	20,723 (31.0)	6117 (27.4)	7632 (34.0)	6970 (31.8)	
		DM, n (%)	24,056 (36.0)	6779 (30.4)	8009 (35.6)	9262 (42.2)	
	AMI, n (%)		15,788 (23.6)	4512 (19.8)	3809 (16.7)	7420 (35.1)	<0.0001
	HT, n (%)		37,614 (56.3)	12,101 (53.1)	13,148 (57.6)	12,324 (58.3)	<0.0001
	CHF, n (%)		9933 (14.9)	2423 (10.6)	2654 (11.6)	4835 (22.9)	<0.0001
	CKD, n (%)		13,734 (20.5)	2757 (12.1)	4276 (18.7)	6689 (31.6)	<0.0001
	AF, n (%)		3005 (4.5)	1060 (4.6)	967 (4.2)	975 (4.6)	0.068
	Stroke, n (%)		4141 (6.2)	1240 (5.4)	1366 (6.0)	1531 (7.2)	<0.0001
	Hyperlipemia, n (%)		43,135 (64.5)	13,524 (59.3)	14,483 (63.5)	15,078 (71.3)	<0.0001
Procedure							
	PCI, n (%)		48,050 (71.9)	14,544 (63.8)	16,533 (72.5)	16,919 (80.0)	<0.0001
	CABG, n (%)		104 (0.2)	24 (0.1)	38 (0.2)	42 (0.2)	0.0407
	DES, n (%)		45,842 (68.6)	13,855 (60.8)	15,772 (69.2)	16,164 (76.4)	<0.0001
	BMS, n (%)		1048 (1.6)	267 (1.2)	359 (1.6)	422 (2.0)	<0.0001
Laboratory tests							
	eGFR, mL/min/1.73 $m^{2}$		78.5 (25.8)	84.2 (22.6)	79.3 (24.9)	71.6 (28.4)	<0.0001
	HGB, g/L		133.5 (17.4)	138.0 (15.9)	134.4 (16.2)	127.7 (18.6)	<0.0001
	SCr, mg/dL		1.0 [0.8, 1.1]	0.9 [0.8, 1.1]	0.9 [0.8, 1.1]	1.0 [0.8, 1.3]	<0.0001
	LDLC, mmol/L		2.9 (1.0)	2.9 (1.1)	2.9 (1.0)	2.9 (1.0)	0.2722
	HDLC, mmol/L		1.0 (0.3)	1.1 (0.3)	1.0 (0.3)	0.9 (0.3)	<0.0001
	HbA1c, %		6.6 (1.4)	6.4 (1.3)	6.5 (1.3)	6.8 (1.6)	<0.0001
	FIB, g/L		3.9 (1.3)	2.8 (0.4)	3.7 (0.4)	5.3 (1.1)	<0.0001
	ALB, g/L		37.6 (4.5)	40.5 (3.7)	37.8 (3.3)	34.2 (4.1)	<0.0001
	FAR		0.108 (0.045)	0.070 (0.010)	0.098 (0.008)	0.158 (0.044)	<0.0001
Medications							
	Beta blocker, n (%)		51,171 (80.0)	16,657 (77.7)	17,722 (80.4)	16,733 (82.1)	<0.0001
	Statins, n (%)		61,237 (95.8)	20,586 (96.0)	21,126 (95.9)	19,450 (95.4)	0.0047
	CCB, n (%)		14,745 (23.1)	5395 (25.2)	5065 (23.0)	4261 (20.9)	<0.0001
	ACEI/ARB, n (%)		44,850 (70.1)	14,386 (67.1)	15,763 (71.5)	14,651 (71.9)	<0.0001
	Diuretics, n (%)		11,602 (18.1)	3112 (14.5)	3547 (16.1)	4929 (24.2)	<0.0001
	Antiplatelet, n (%)		62,188 (97.3)	20,788 (97.0)	21,444 (97.3)	19,885 (97.5)	0.0016
Clinical Outcomes							
	All-cause mortality, n (%)		10,534 (15.8)	2090 (9.4)	3237 (14.4)	5204 (23.7)	<0.0001
	Cardiovascular mortality, n (%)		4991 (7.5)	833 (3.7)	1378 (6.1)	2779 (12.7)	<0.0001
	Cancer mortality, n (%)		1092 (1.6)	193 (0.9)	365 (1.6)	533 (2.4)	<0.0001

Abbreviations: NGR, normal glucose regulation; PreDM, prediabetes mellitus; DM, diabetes mellitus; AMI, acute myocardial infarction; HT, hypertension; CHF, congestive heart failure; CKD, chronic kidney disease; AF, atrial fibrillation; PCI, percutaneous coronary intervention; CABG, coronary artery bypass grafting; DES, drug eluting stent; BMS, bare metal stent; eGFR, estimated glomerular filtration rate; HGB, hemoglobin; SCr, serum creatinine; LDLC, low density lipoprotein cholesterol; HDLC, high density lipoprotein cholesterol; HbA1c, hemoglobin A1c; FIB, fibrinogen; ALB, albumin; FAR, fibrinogen-to-albumin ratio; CCB, calcium channel blocker; ACEI/ARB, angiotensin-converting enzyme inhibitor/angiotensin receptor blocker; FAR-L, low FAR; FAR-M, median FAR; FAR-H, high FAR.

During a median follow-up of 4.68 years, 10,534 (15.8%) participants died, of which, 4991 (7.5%) were cardiovascular-specific deaths, and 1092 (1.6%) were cancer-specific deaths. For all-cause death or cause-specific death, the proportion of patients with a higher FAR was significantly higher than the FAR-L group (Table 1). A similar trend was also observed in patients with different glucose metabolism status (Table 2).

**Table 2.** **Multivariable Cox regression models of the relationship between FAR and clinical outcomes in patients with different glucose metabolism**.

FAR		All-cause mortality		Cardiovascular mortality		Cancer mortality	
		Events/subjects	HR (95% CI)	Events/subjects	HR (95% CI)	Events/subjects	HR (95% CI)
NGR		3125/21,982		1395/21,982		315/21,982	
	FAR per-SD increase		1.12 (1.09–1.16) **		1.19 (1.14–1.25) **		1.22 (1.11–1.34) **
	FAR-L	852/9439	Ref.	349/9439	Ref.	66/9439	Ref.
	FAR-M	988/6825	1.18 (1.08–1.29) **	405/6825	1.15 (1.00–1.33)	111/6825	1.69 (1.25–2.28) **
	FAR-H	1283/5715	1.35 (1.24–1.48) **	640/5715	1.47 (1.28–1.68) **	138/5715	2.02 (1.50–2.73) **
PreDM		3026/20,723		1238/20,723		386/20,723	
	FAR per-SD increase		1.18 (1.14–1.21) **		1.23 (1.18–1.29) **		1.25 (1.14–1.36) **
	FAR-L	552/6117	0.86 (0.78–0.96) *	179/6117	0.68 (0.57–0.81) **	62/6117	1.21 (0.87–1.70)
	FAR-M	998/7632	0.99 (0.90–1.08)	381/7632	0.88 (0.76–1.02)	132/7632	1.61 (1.20–2.16) *
	FAR-H	1476/6970	1.32 (1.21–1.44) **	678/6970	1.34 (1.17–1.53) **	192/6970	2.27 (1.70–3.02) **
DM		4383/24,056		2358/24,056		391/24,056	
	FAR per-SD increase		1.21 (1.18–1.25) **		1.28 (1.24–1.33) **		1.25 (1.14–1.37) **
	FAR-L	705/6029	1.01 (0.91–1.11)	314/6029	1.07 (0.92–1.25)	66/6029	1.24 (0.88–1.74)
	FAR-M	1283/8167	1.21 (1.11–1.32) **	610/8167	1.29 (1.13–1.47) **	127/8167	1.59 (1.18–2.15) *
	FAR-H	2395/8959	1.71 (1.58–1.86) **	1434/8959	2.11 (1.86–2.38) **	198/8959	2.20 (1.66–2.92) **

Adjusted for age, gender, hypertension, acute myocardial infarction, stroke, chronic kidney disease, hyperlipemia, congestive heart failure. FAR, fibrinogen-to-albumin ratio; NGR, normal glucose regulation; PreDM, prediabetes mellitus; DM, diabetes mellitus; FAR-L, low FAR; FAR-M, median FAR; FAR-H, high FAR; HR, hazard ratio; CI, confidence interval; SD, standard deviation. *, *p* < 0.05; **, *p* < 0.001.

### 3.2 Different Glucose Metabolism Status, FAR, and Clinical Outcomes

**Fig. 2.**
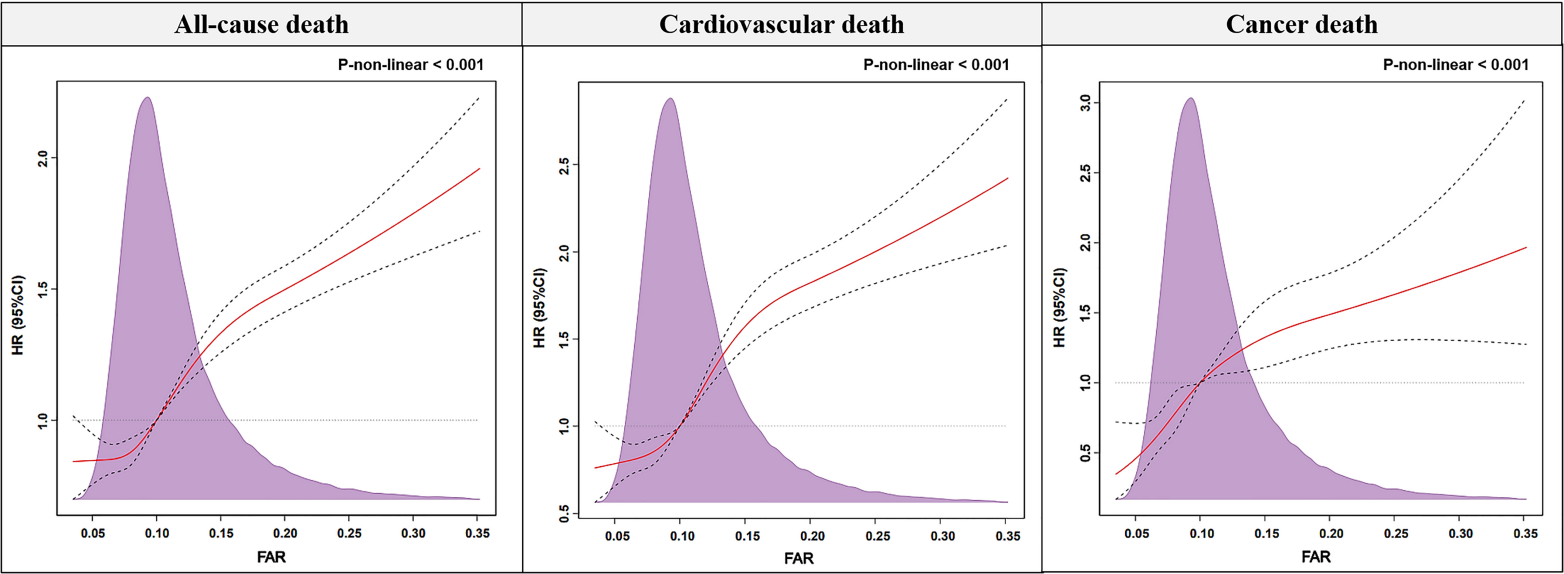
**Restricted spline curve for the association between FAR and all-cause, cardiovascular, and cancer mortality based on multivariate Cox regression models**. The multivariate Cox regression model includes adjustment for age, gender, hypertension, acute myocardial infarction, stroke, chronic kidney disease, hyperlipemia, and congestive heart failure. FAR, fibrinogen-to-albumin ratio; HR, hazard ratio.

**Fig. 3.**
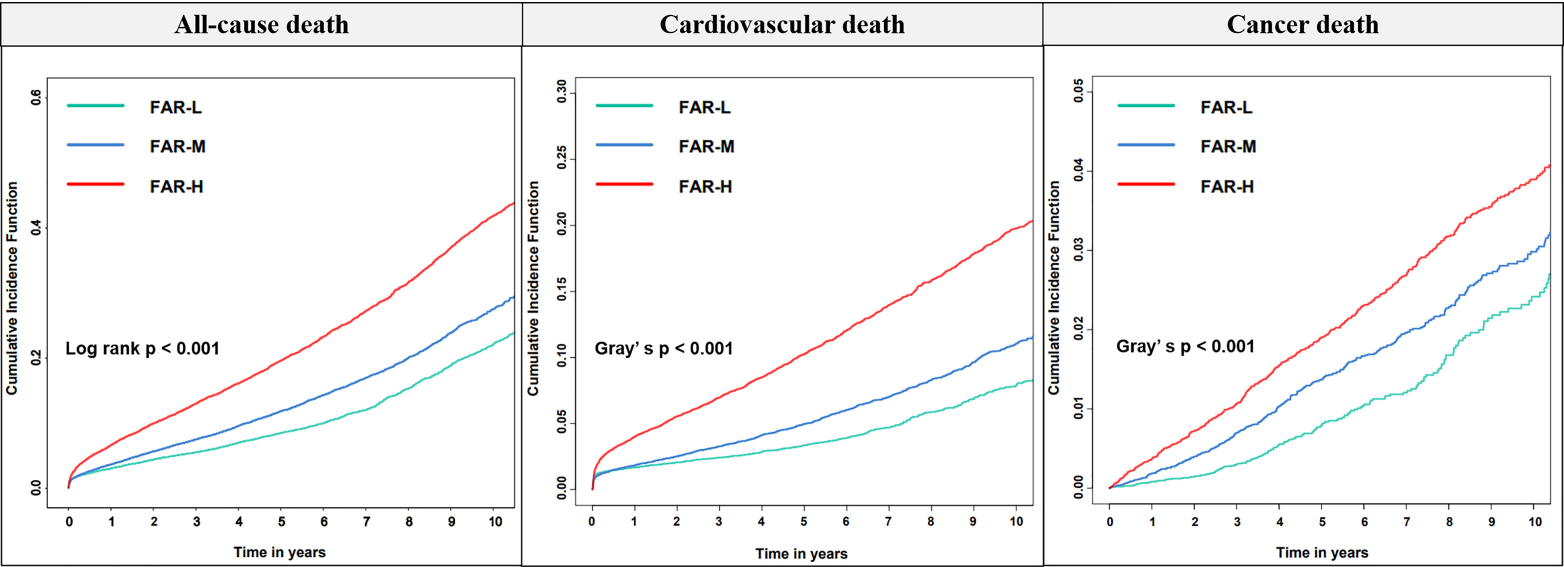
**Kaplan–Meier curves for all-cause, cardiovascular, and cancer mortality according to different FAR levels based on univariate Cox regression models**. FAR, fibrinogen-to-albumin ratio; FAR-L, low FAR; FAR-M, median FAR; FAR-H, high FAR.

**Fig. 4.**
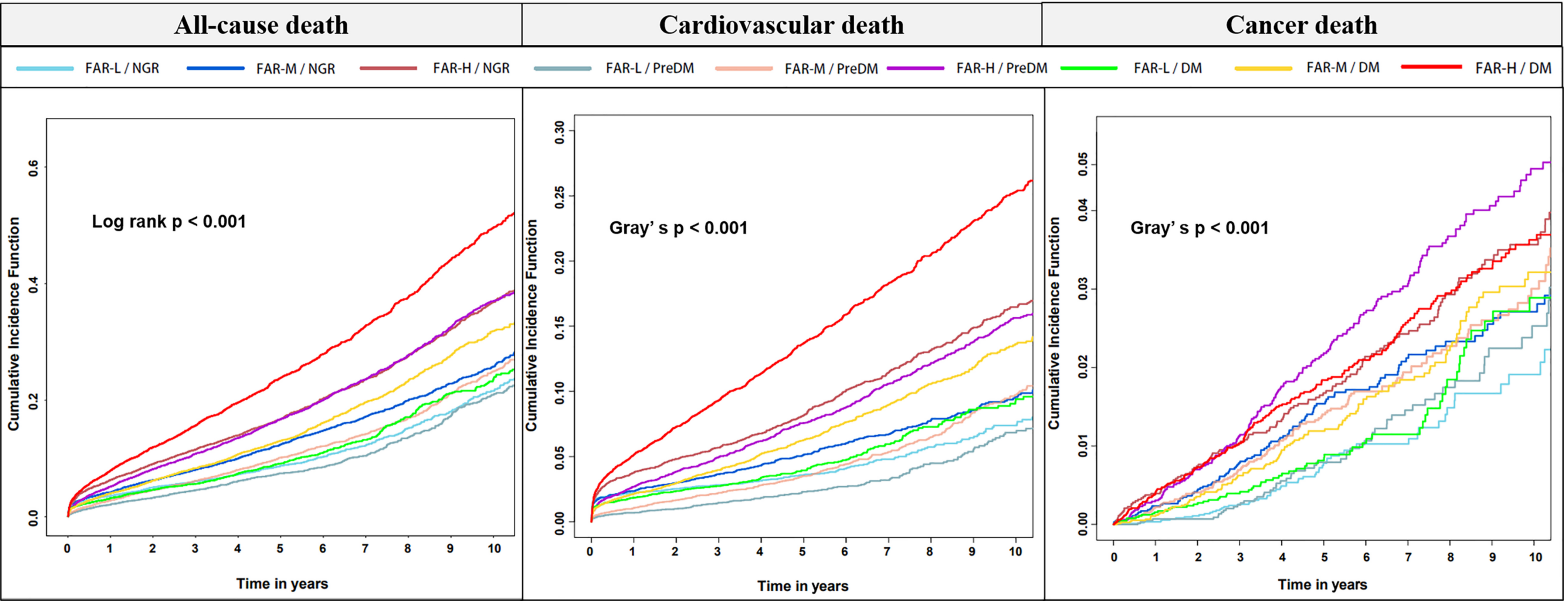
**Kaplan–Meier curves for all-cause, cardiovascular, and cancer mortality according to states of both FAR levels and glucose metabolism based on univariate Cox regression models**. FAR, fibrinogen-to-albumin ratio; FAR-L, low FAR; FAR-M, median FAR; FAR-H, high FAR; NGR, normal glucose regulation; PreDM, prediabetes mellitus; DM, diabetes mellitus.

RCS analysis demonstrated that the risk of all-cause or cause-specific death increased as FAR increased in CAD patients, but the relationship was not linear (all nonlinear *p* < 0.001) (Fig. 2). Kaplan-Meier curves indicated that the FAR-H group had the highest risk of all-cause mortality. The risk of cause-specific mortality also showed similar results (Fig. 3). In the FAR level as well as different glucose metabolism states, CAD patients were further classified into 9 groups. DM Patients in FAR-H had the highest risk of all-cause mortality and cardiovascular mortality, while the risk of cancer mortality was highest in FAR-H with PreDM (Fig. 4).

Table 2 shows three Cox regression models to further investigate the association among FAR, different glucose metabolism states and clinical outcomes. After adjusting for age, gender, HT, CKD, stroke, AMI, hyperlipemia, CHF, the higher FAR group had a higher risk for all-cause and cause-specific mortality in NGR patients; similar results were found in the PreDM and DM groups. The risks of all-cause mortality (NGR, PreDM, DM: HR (95% CI) = 1.12 (1.09–1.16), 1.18 (1.14–1.21), 1.21 (1.18–1.25), respectively; all *p* values < 0.001), cardiovascular mortality (NGR, PreDM, DM: HR (95% CI) = 1.19 (1.14–1.25), 1.23 (1.18–1.29), 1.28 (1.24–1.33), respectively; all *p* values < 0.001) and cancer mortality (NGR, PreDM, DM: HR (95% CI) = 1.22 (1.11–1.34), 1.25 (1.14–1.36), 1.25 (1.14–1.37), respectively; all *p* values < 0.001) elevated with per 1-SD increase in FAR. The risk increased with decreased levels of glucose metabolism (all *p *for trend <0.001). In addition, FAR-H with DM group had the highest risk of all-cause and cardiovascular mortality (HR (95% CI) = 1.71 (1.58–1.86), HR (95% CI) = 2.11 (1.86–2.38), respectively; all *p* values < 0.001). FAR-H with PreDM group was significantly associated with the highest risk of cancer mortality (HR (95% CI) = 2.27 (1.70–3.02), *p* value < 0.001).

### 3.3 Risk Prediction for Clinical Outcomes with Different Glucose Metabolism Status 

In order to determine whether FAR has an additional predictive value compared with the original model, we calculated the C-indexes with and without FAR. The original model included age, gender, HT, CHF, AMI, CKD, stroke and hyperlipemia. Regardless of all-cause or cause-specific mortality, the addition of FAR to the model resulted in a significant improvement in C-indexes for NGR, PreDM and DM patients (all *p* value < 0.001) (Table 3).

**Table 3.** **C-index of FAR for predicting clinical outcomes in subjects with different glucose metabolism status**.

Model	All-cause mortality		Cardiovascular mortality		Cancer mortality	
	C-index	*p*	C-index	*p*	C-index	*p*
NGR original model	0.682 (0.670–0.694)		0.720 (0.704–0.736)		0.720 (0.693–0.747)	
NGR original model + FAR	0.687 (0.675–0.699)	<0.001	0.724 (0.708–0.74)	<0.001	0.733 (0.706–0.760)	<0.001
PreDM original model	0.673 (0.661–0.685)		0.748 (0.732–0.764)		0.701 (0.676–0.726)	
PreDM original model + FAR	0.683 (0.671–0.695)	<0.001	0.758 (0.742–0.774)	<0.001	0.721 (0.697–0.745)	<0.001
DM original model	0.691 (0.681–0.701)		0.734 (0.722–0.746)		0.685 (0.656–0.714)	
DM original model + FAR	0.701 (0.693–0.709)	<0.001	0.748 (0.736–0.760)	<0.001	0.700 (0.673–0.727)	<0.001

Original model included age, gender, hypertension, congestive heart failure, acute myocardial infarction, chronic kidney disease, stroke, hyperlipemia. FAR, fibrinogen-to-albumin ratio; NGR, normal glucose regulation; PreDM, prediabetes mellitus; DM, diabetes mellitus.

### 3.4 Association between FAR and Glucose Metabolism

FAR showed a significantly positive correlation with glucose metabolism indexes including HbA1c (r = 0.14, *p* < 0.001) and FBG (r = 0.10, *p* < 0.05). **Supplementary Fig. 1** shows all correlations between HbA1c, FBG, FAR and its components.

## 4. Discussion

In this retrospective study, we demonstrated that elevated FAR was significantly related to a higher risk of all-cause, cardiovascular, and cancer mortality in CAD patients with different glucose metabolism status. Furthermore, abnormal glucose metabolism increases this association. In addition, adding FAR to the original model enhanced the predictive power for long-term mortality. Our results indicate that FAR is an effective risk stratification tool for CAD patients with NGR, PreDM and DM.

Abnormal glucose metabolism is common in patients with CAD [4]. In a diabetes and heart survey involving 110 centers in Europe, up to 31% of patients with CAD were already known to have diabetes and 40% had newly discovered glucose abnormalities [3]. Hyperglycemia caused by insulin resistance induces mitochondrial dysfunction and endoplasmic reticulum stress, leading to endothelial dysfunction, ROS accumulation and inflammation, which ultimately contributes to the development of CAD [27, 28]. Several epidemiological studies have also demonstrated that abnormal glucose metabolism was related to higher risk of adverse cardiovascular events and mortality among CAD patients. A large cohort study, including 3276 postinfarction patients, indicated that the occurrence of sudden cardiac death was higher in DM patients compared to non-DM patients [29]. Lenzen *et al*. [5] found that CAD patients with previously recognized and newly detected DM was associated with a 1.4- and 1-fold elevated risk of 1-year mortality, respectively, compared to those with NGR. The first Diabetes Mellitus Insulin Glucose Infusion in Acute Myocardial Infarction study also demonstrated an apparent beneficial effect of enhanced insulin-based glucometabolic control after AMI, which prolonged survival by 2.3 years [30]. Therefore, it is critical to monitor the glucose metabolism of CAD patients and intervene as soon as possible to improve the long-term prognosis of high-risk populations.

CAD and DM involve complex physiological processes, and chronic inflammation has been widely accepted as the pathological mechanism for these two diseases [9]. CAD is essentially an inflammatory disease, risk factors such as dyslipidemia, hyperglycemia, smoking, and hypertension induce endothelial cell damage, stimulate immune cell proliferation, resulting in the upregulate of the expression of inflammatory and procoagulant cytokines such as IL-1β and FIB [31, 32, 33, 34], ultimately leading to CAD. In diabetes, hyperglycemia and elevated free fatty acids can activate the JNK/IKK NF-κB pathway and inflammatory cascade, which may trigger insulin resistance to exacerbate the development of diabetes and long-term complications [9, 35, 36]. Previous studies have showed that inflammation biomarkers can be used as an effective tool to predict the poor prognosis of CAD and DM patients. Higher FIB is independently linked to major adverse cardiovascular events in CAD patients, particularly in PreDM and DM [37]. The acute phase inflammatory biomarker albumin has also been found to be strongly associated with cardiovascular outcomes in CAD and DM patients [38, 39, 40]. Fibrinogen-to-albumin ratio (FAR), is a widely used novel inflammatory biomarker that has been shown outstanding capability in predicting the risk of poor clinical outcomes in patients with cancer and cardiovascular disease [41, 42]. Nevertheless, the potential connection between FAR and long-term mortality among CAD patients with different glucose metabolism states, especially in those with PreDM, is not entirely understood. This research, for the first time demonstrates the relationship between FAR and long-term mortality in CAD patients with PreDM. We found that higher FAR levels in both NGR, PreDM and DM patients were significantly correlated with higher risks of all-cause and cause-specific mortality. In addition, the results of this study showed that abnormal glucose metabolism amplifies the relationship between FAR and mortality. The inflammatory state in diabetic patients increases the expression of IL-6 and TNF-α, and these cytokines increase the synthesis of FIB and the degradation of ALB, leading to the increased activation of the coagulation pathway and decreased antiplatelet capacity [17, 43, 44]. These changes will eventually result in the formation of thrombosis *in vivo*, which may be the potential pathophysiological mechanism responsible for this effect.

Several studies have found a strong connection between diabetes and cancer. Diabetes may stimulate the proliferation and metastasis of cancer cells and influence the development of tumors through various biological mechanisms, such as hyperinsulinemia, hyperglycemia or chronic inflammation [45]. Moreover, patients with diabetes have a higher cancer mortality compared with non-diabetic patients [46, 47]. Therefore, this study also evaluated the predictive value of FAR for cancer death in CAD patients with different glucose metabolism states. Consistently, our research showed that PreDM and DM patients with FAR-H were significantly linked to higher risks of cancer mortality. However, the connection between FAR and cancer mortality in CAD patients classified by different glucose metabolism states is not fully understood. Therefore, additional research is required to clarify these potential connections.

In our study, elevated FAR was associated with increased long-term mortality in patients with CAD, and correlated with different glucose metabolism states. Abnormal glucose metabolism amplifies the relationship between FAR and death. Moreover, to the best of our knowledge, this study revealed the predictive effect of FAR on mortality risk in CAD patients with prediabetes, for the first time. On the basis of the correlation between FAR and mortality risk, monitoring FAR levels and controlling inflammation with several anti-inflammatory therapy may play an important role in decreasing mortality in high-risk groups. Further investigations will be necessary to prospectively verify the prediction power of FAR for all-cause, cardiovascular and cancer mortality among CAD patients with different glucose metabolism status.

This research has several limitations. First, this research was a retrospective observational analysis, which didn’t reflect direct causation. Second, due to data limitations, the effect of the severity of coronary artery disease on the relationship between FAR and mortality was not analyzed. Third, we only assessed FAR levels at the time of admission. The dynamic changes of FAR were absent during follow-up. Fourth, despite the adjustment for potential confounding factors, possible confounders could not be fully adjusted. Fifth, this study only included CAD patients in China, and whether the findings of this study can be generalized to other populations remains unclear.

## 5. Conclusions

In conclusion, FAR is a valuable tool for risk stratification of CAD patients with NGR, PreDM, and DM. Increased levels of FAR were significantly associated with higher risks of all-cause and cause-specific mortality. Moreover, abnormal glucose metabolism amplifies the relationship between FAR and mortality. FAR levels can provide more precise risk stratification for high-risk CAD patients and provide essential information for improving the long-term prognosis of these patients.

## Data Availability

The datasets generated and analyzed during the current study are not publicly available due to the institution policy but are available from the corresponding author on reasonable request.

## Abbreviations

CAD, coronary artery disease; DM, diabetes mellitus; PreDM, prediabetes mellitus; NGR, normal glucose regulation; AMI, acute myocardial infarction; HT, hypertension; CHF, congestive heart failure; CKD, chronic kidney disease; AF, atrial fibrillation; PCI, percutaneous coronary intervention; CABG, coronary artery bypass grafting; DES, drug eluting stent; BMS, bare metal stent; eGFR, estimated glomerular filtration rate; HGB, hemoglobin; SCr, serum creatinine; LDLC, low density lipoprotein cholesterol; HDLC, high density lipoprotein cholesterol; HbA1c, Hemoglobin A1c; FIB, fibrinogen; ALB, albumin; FAR, fibrinogen-to-albumin ratio; CCB, calcium channel blocker; ACEI/ARB, angiotensin-converting enzyme inhibitor/angiotensin receptor blocker; FAR-L, low FAR; FAR-M, median FAR; FAR-H, high FAR.

## Supplementary Material

Supplementary material associated with this article can be found, in the online version, at https://doi.org/10.31083/j.rcm2411317.
