# Supplementary figures and images for: Fibrinogen-to-Albumin Ratio and Long-Term Mortality in Coronary Artery Disease Patients with Different Glucose Metabolism Status
Source: Rev Cardiovasc Med. 2023 Nov 16;24(11):317. doi: 10.31083/j.rcm2411317 (PMC11272855; doi:10.31083/j.rcm2411317)

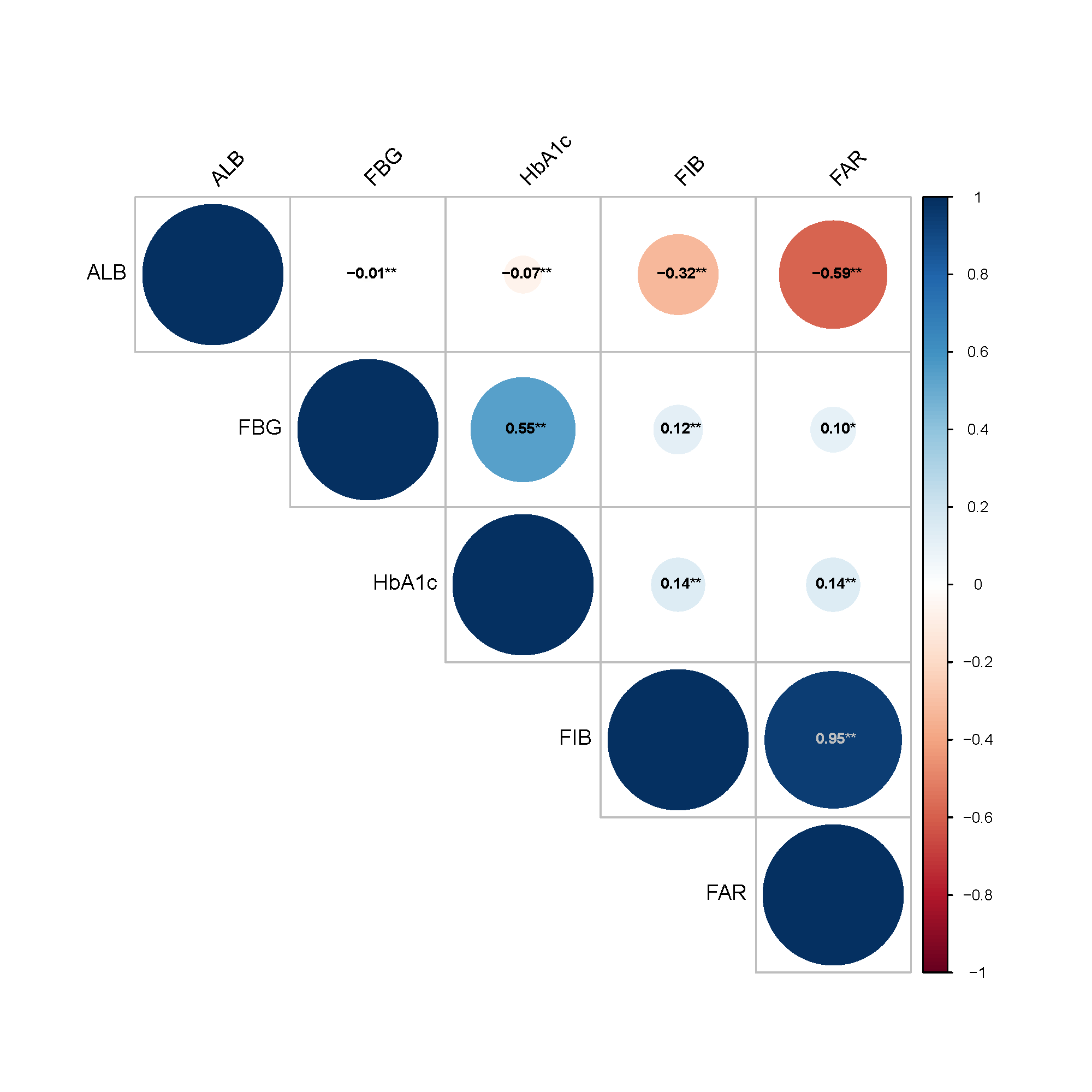

Supplement: Supplementary file 1 [file 2153-8174-24-11-317-s1.tif]
